# A Gold Crown with Perfect Articulating Surface

**Published:** 1892-05

**Authors:** L. C. Bryan

**Affiliations:** Basel, Switzerland


### A GOLD CROWN WITH PERFECT ARTICULATING SURFACE.[1]

BY DR. L. C. BRYAN, BASEL, SWITZERLAND.

THE gold crown, models of which are presented in the various stages of its construction, is very simply made, and accomplishes more successfully the objects of a gold crown for all varying and irregular cases than those made in rigid forms or stamped up by one of any number of dies. No set of dies, even the forty of which Dr. Miller presented to this meeting, will give a perfect articulating surface to the antagonizing teeth in all cases, and the outfit usually necessary to one who will undertake crown-work is so large and complicated that the busy practitioner is inclined to consider the undertaking too great to become familiar with its details, and add to his stock of laboratory tools an additional outfit with which he may not succeed. The Mellotte moulding-box is the only outfit required in addition to the usual laboratory tools for metal work for this crown.

The root is prepared, treated, and filled in the usual way, after which an impression and "bite" are taken and models made and mounted in the articulator. For this purpose the crown impression-cup of Ash & Sons, passed around, is the best, as the root and adjoining teeth and the antagonists are all taken in one cup and can be set in the crown articulator, securing an immediate bite, adjusted and ready to proceed.

The method of Mr. Parris, given in Ash & Sons' *Quarterly* for June, 1891, of filling soft copper amalgam into the impression before pouring the body of the model in plaster, where much use is to be made of the models, as in trying in rings, fitting clamps, etc., is to be highly recommended. The articulated models being ready, the plaster at the gum is cut away around the root to the depth it is desired to fit the band or ring below the gum margin. The ring is made and adjusted to the model as high as the bite will allow, filed flat on its upper surface with a broad file, and filled in with modelling composition. The ring and its composition filling may now be tried in the mouth, if convenient, and the bite taken in this way or the crown be finished on the models. In the latter case, with the ring and its soft composition in place on the bite, model in

---

[1] Read before the American Dental Society of Europe, at Heidelberg, August 4, 1891.

the articulator. The models are closed and the antagonizing tooth or teeth pressed into the composition to indicate the bite. The composition is then artistically carved away to represent the desired masticating surface with its sulci and cusps, care being taken to have the surface of the carved composition-crown just so far removed from the antagonizing tooth as the thickness of the gold plate used for the surface cap. An impression of this composition crown-cap is now taken in mouldine, which is simply a kind of artist's clay mixed with glycerin. The metal accompanying the mouldine is fused in a spoon over a gas or spirit lamp, and is poured into the clay impression. This model represents the free edge of the ring and the carved surface of the desired crown. A sheet of crown or pure gold is now laid on a block of lead and this metal die hammered into it until the desired cusps are formed. This is now trimmed and the resulting cap fitted on the ring over the composition and a good joint secured. The ring, cap, and composition are now removed and bound solidly with fine iron binding-wire.

The composition being removed from the ring, the ring and cap are soldered firmly together, the cusps in the cap being filled level full of solder to give strength and durability to the crown's masticating surface.

In the carving of the composition, to represent the desired crown surface, there is an opportunity for the display of high artistic skill in reproducing the lost crown while conforming to the articulation, or the same success may be attained in securing a perfect articulation by the most unskilful carver.

